# The Arabic Version of the Mobile App Rating Scale: Development and Validation Study

**DOI:** 10.2196/16956

**Published:** 2020-03-03

**Authors:** Marco Bardus, Nathalie Awada, Lilian A Ghandour, Elie-Jacques Fares, Tarek Gherbal, Tasnim Al-Zanati, Stoyan R Stoyanov

**Affiliations:** 1 Department of Health Promotion and Community Health Faculty of Health Sciences American University of Beirut Beirut Lebanon; 2 Department of Epidemiology and Population Health Faculty of Health Sciences American University of Beirut Beirut Lebanon; 3 Department of Nutrition and Food Sciences Faculty of Agricultural and Food Sciences American University of Beirut Beirut Lebanon; 4 University Sports Office of Student Affairs American University of Beirut Beirut Lebanon; 5 International Medical Corps Amman Jordan; 6 School of Design Creative Industries Faculty Queensland University of Technology Brisbane Australia

**Keywords:** validation studies as topic, mHealth, mobile app rating scale, Arab world, eHealth, app quality, app evaluation, mobile app

## Abstract

**Background:**

With thousands of health apps in app stores globally, it is crucial to systemically and thoroughly evaluate the quality of these apps due to their potential influence on health decisions and outcomes. The Mobile App Rating Scale (MARS) is the only currently available tool that provides a comprehensive, multidimensional evaluation of app quality, which has been used to compare medical apps from American and European app stores in various areas, available in English, Italian, Spanish, and German. However, this tool is not available in Arabic.

**Objective:**

This study aimed to translate and adapt MARS to Arabic and validate the tool with a sample of health apps aimed at managing or preventing obesity and associated disorders.

**Methods:**

We followed a well-established and defined “universalist” process of cross-cultural adaptation using a mixed methods approach. Early translations of the tool, accompanied by confirmation of the contents by two rounds of separate discussions, were included and culminated in a final version, which was then back-translated into English. Two trained researchers piloted the MARS in Arabic (MARS-Ar) with a sample of 10 weight management apps obtained from Google Play and the App Store. Interrater reliability was established using intraclass correlation coefficients (ICCs). After reliability was ascertained, the two researchers independently evaluated a set of additional 56 apps.

**Results:**

MARS-Ar was highly aligned with the original English version. The ICCs for MARS-Ar (0.836, 95% CI 0.817-0.853) and MARS English (0.838, 95% CI 0.819-0.855) were good. The MARS-Ar subscales were highly correlated with the original counterparts (*P*<.001). The lowest correlation was observed in the area of usability (*r*=0.685), followed by aesthetics (*r*=0.827), information quality (*r*=0.854), engagement (*r*=0.894), and total app quality (*r*=0.897). Subjective quality was also highly correlated (*r*=0.820).

**Conclusions:**

MARS-Ar is a valid instrument to assess app quality among trained Arabic-speaking users of health and fitness apps. Researchers and public health professionals in the Arab world can use the overall MARS score and its subscales to reliably evaluate the quality of weight management apps. Further research is necessary to test the MARS-Ar on apps addressing various health issues, such as attention or anxiety prevention, or sexual and reproductive health.

## Introduction

### Background

Preventing noncommunicable diseases (NCDs) is a major public health priority [1], globally and in the Arab region, where heart disease, diabetes, hypertension, stroke, and other cardiovascular disorders are commonly observed in both low-income and high-income countries [2]. The prevalence of overweight ranged from 19% to 57% in the Middle East and North Africa (MENA) region, and from 6% to 53% in the Eastern Mediterranean area [3], but it reached higher levels in high-income countries of the Gulf, such as Kuwait and the United Arab Emirates [4]. Similar trends are observed for type 2 diabetes (an estimated 9% of the population), which is projected to affect 60 million Arabs in 2030 [5].

Mobile apps provide a unique opportunity to address NCDs worldwide [6,7], as these technologies are available among both high- and low-income populations [8]. In the world, there are more than 7 billion mobile subscribers [9] (3.4 billion of whom are mobile phone users) [10]. Recent systematic reviews provide some evidence of the efficacy of mobile health (mHealth) apps for promoting dietary self-regulation [11] and weight management [12-18]. In 2017, there were more than 350,000 mHealth apps available in Web-based stores [19], offering a wide variety of services for primary or secondary prevention [20]. The global health app market was worth US $25 billion in 2017 and US $37 billion in 2019, and it is projected to reach US $72 billion in 2020 [21]. In the Arab world, the mHealth market is also rapidly growing and is expected to reach US $1.3 billion by 2019 [22]. However, the market is extremely volatile and unstable; in some cases, app turnover can be 3.7 days in Google Play (for Android phones) and 13.7 days in App Store (for iOS phones) over 9 months [23]. Some research shows that many apps are downloaded less than 500 times, or never used [24]. Qualitative studies show that users stop using health apps because of hidden costs, increased data entry burden [25], and low engagement [26]. From a content point of view, apps generally lack evidence-based and theoretical support [27,28]. The instability and unpredictability of the health app market pose several challenges for both experts (ie, health professionals and researchers) and laypersons (ie, customers, end users, and patients), who need appropriate tools to decide which apps are worth using and recommending.

Evaluating app quality has become a fundamental task for researchers, as the failure to accurately and adequately evaluate health app quality might compromise end users’ well-being and decrease their confidence in the technology [23]. Various frameworks and tools exist to evaluate app quality [29], but they generally lack multidimensionality and cultural flexibility, focusing on either information content, functionality, usability, accountability, impact, or popularity dimensions [29,30].

The Mobile App Rating Scale (MARS) [31] is a multidimensional comprehensive tool for assessing the quality of mHealth apps for experts. According to the scale developers, MARS includes 19 questions or items, which have been logically grouped according to *objective* dimensions of engagement (five items), functionality (four items), aesthetics (three items), and information quality (seven items). The instrument also includes four items that are deemed more *subjective* as they include questions such as the following: “Would you recommend this app to people who might benefit from it?” “How many times do you think you would use this app in the next 12 months if it was relevant to you?” “Would you pay for this app?” and “What is your overall 5-star rating of the app?”

In the development of MARS, the authors involved a multidisciplinary team of designers, health professionals, and developers [31], making the scale user friendly, dependable, and broadly applicable to different health apps. MARS has been used by trained raters to evaluate apps addressing a wide range of behaviors and health-related issues, such as drunk-driving prevention [32], speech sound disorders [33], self-care management of heart failure symptoms [34], mental health and mindfulness [35], quality of life [36], weight loss and smoking cessation [37], or weight management, including physical activity and calorie counting apps [38]. A simplified version for end users (user version of the MARS, uMARS) has also been developed [39]; it includes the same domains of the MARS tool, using simplified language and omitting items that would require rater expertise, so that it can be used without training and by laypersons or end users [31].

The MARS tool has been recently translated into Italian [40], Spanish [41], and German [42], and there are ongoing projects for translating it into nine other languages. However, there is currently no instrument for assessing the quality of health apps in Arabic. The Arab world geographically includes Africa (Algeria, Comoros, Djibouti, Egypt, Libya, Mauritania, Morocco, Somalia, Sudan, and Tunisia), Middle East, and parts of Asia (Bahrain, Iraq, Jordan, Kuwait, Lebanon, Oman, Palestine/Israel, Qatar, Saudi Arabia, Syria, the United Arab Emirates, and Yemen). Even though the original MARS tool could be used by Arabs who are also fluent in English, the majority of people living in the MENA region have “very low” English proficiency, according to the Education First English Proficiency Index [43]. With a growing mHealth market in the Arab world, along with growing public health concerns about NCD trends in the region, there is an urgent need for tools such as MARS to be available for Arabic-speaking health professionals and end users in the region.

### Objectives

This study aimed to fill the gap by adapting the MARS in Arabic (MARS-Ar) and validating the instrument with a sample of popular weight management apps, available in the category “Health and Fitness” in the app stores of the Arab world.

## Methods

### Study Design Overview

This study followed a well-established and so called “universalist approach” [44], which is based on the assumption that an individual’s response to any given question or concept depends on the individual’s culture [45]. We followed a similar procedure used by researchers who developed and validated the MARS tool in Italian [40] and German [42]. This process comprises several phases, including (1) translation and cultural adaptation with back-translation, (2) review, (3) piloting, and (4) validation or psychometric evaluation. The local Institutional Review Board approved the study protocol and research procedures involving human subjects on November 1, 2018 (ref. nr: SBS-2018-0394). In the section below, we describe the process of translation and cultural adaptation, including the review and piloting phases. In the results section, we describe the results of the validation or psychometric evaluation of the MARS-Ar tool.

### Phase 1: Translation and Cultural Adaptation Process

**Figure 1:**
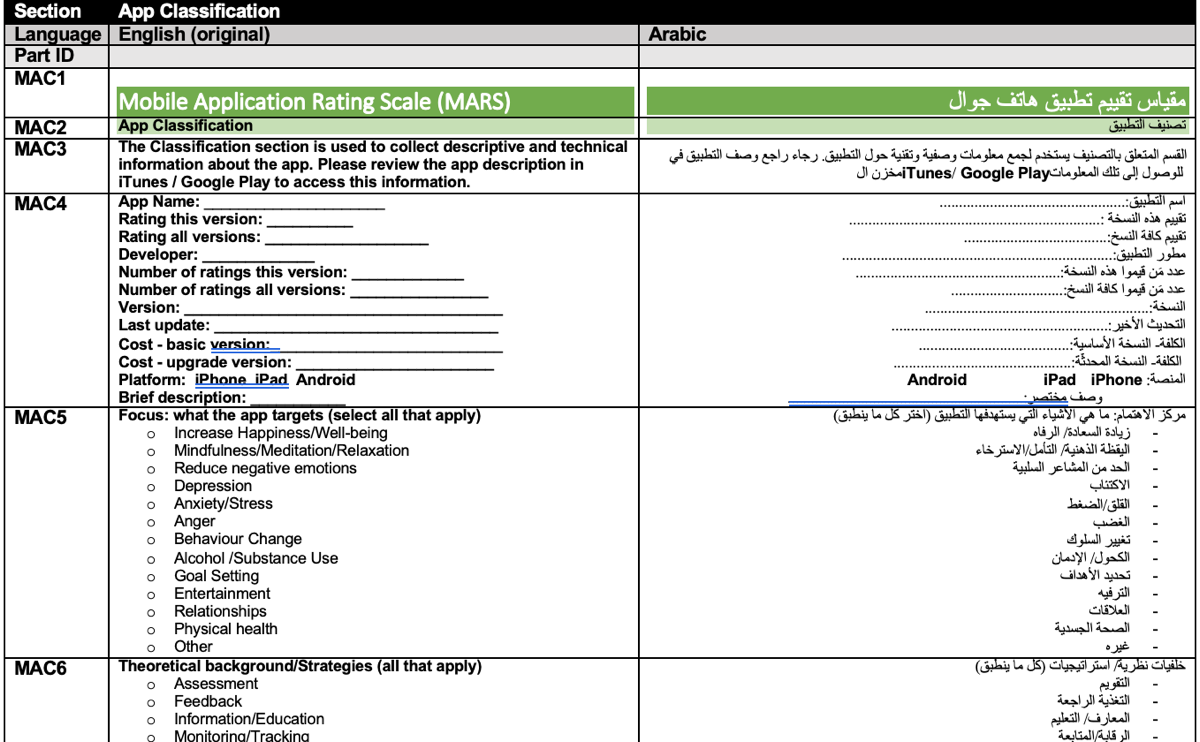
Format of the document used in the Mobile App Rating Scale-Arabic translation process.

The MARS tool was first translated in Arabic by a professional English-Arabic translator, with expertise in technological topics, who was recruited from a pool of contractors of the American University of Beirut. The translated instrument was broken down into sections and parts, including titles, introductory paragraphs and instructions, and the actual MARS items, with several answer options. MARS was segmented into 59 parts; the translated parts were laid out in a table with the original English version. Each segment was associated with a unique identifier (see Figure 1) so that it would be easier to identify any editing modifications and quantitative ratings for the translation provided by experts.

### Phase 2: Review

The review phase comprised two rounds of Web-based consultations among Arabic-speaking experts from various academic and governmental institutions in the MENA region, who responded to an initial call for Arabic-speaking academics (language experts, social scientists, computer scientists, and engineers), practitioners, or app developers who would be willing to evaluate and provide feedback on the Arabic translation of MARS.

#### Recruitment

The research team members sent email invitations to their personal social networks and to the Public Health in the Arab World mailing list, a subscription-based email list that focuses on issues related to public health in the Arab World and includes more than 1900 subscribers worldwide. The call was also shared on professional social networking sites (eg, LinkedIn and ResearchGate) and on the research team members’ personal social media profiles on Facebook and Twitter. The email and the social media posts contained a link to a consent form, stored on MailChimp servers, where interested participants provided consent for participation in the study.

Between March 26, 2019, and April 17, 2019, 19 Arabic-speaking experts from various academic and governmental institutions responded to the call and agreed to participate in the translation and cultural adaptation phase of the project. Participants included 9 representatives from Lebanon (the Ministry of Public Health, the American University of Beirut, the Lebanese American University, and a local Nongovernmental Organization), 2 representatives from Egypt (Alexandria Regional Centre for Women’s Health and Development and Egypt Health Foundation), 2 representatives from Jordan (King Hussein Cancer Center and a tech company ISEET), and 1 representative each from Syria (Action Against Hunger), Morocco (Faculty of Sciences, University Ibn Tofail, Kénitra), Qatar (Hamad Bin Khalifa University-College of Science and Engineering), Saudi Arabia (Saudi Center for Disease Control and Prevention), the United Arab Emirates (Specialized rehabilitation hospital and Capital Health), and the United States (Wayne State University).

#### Review Consultation Procedures

The research team set up a Web-based consultation system based on email communications through MailChimp, Google Docs, and a Web-based survey hosted on the American University of Beirut servers (LimeSurvey, GmbH) [46]. Enrolled experts received an email with a Word document containing the translation and original version of the MARS tool, as shown in Figure 1. The experts were instructed to (1) download the Word document on their computer, (2) add comments and edits to the file using “track changes,” (3) upload the edited document on LimeSurvey using personalized credentials, and (4) complete an evaluation form rating the translation for each part. Experts were asked to rate the appropriateness and accuracy of each segment using 5-point Likert-type scales (5=very appropriate, 1=very inappropriate and 5=very accurate, 1=very inaccurate). As the MARS instrument was segmented into 59 parts, each expert expressed a total of 118 ratings.

Out of the 19 available experts, 14 experts (14/19, 74% response rate) provided editing suggestions and completed the Web-based form evaluating the appropriateness and accuracy of the translated parts. An analysis of the Excel “comment dashboard” showed that experts provided a total of 287 editing suggestions for the MARS. In all, 3 reviewers provided editing suggestions for more than 50% of the MARS parts; 5 reviewers provided suggestions for more than 30%, and 6 reviewers provided suggestions for less than 30%. The parts that received the most editing suggestions (ie, from 10 to 14 reviewers) were the “Theoretical background/Strategies” and the “Technical aspects of app” in the “App Classification” section, followed by MARS item number 1, that is, “Entertainment” (*Is the app fun/entertaining to use? Does it use any strategies to increase engagement through entertainment, for example, through gamification?*), the description of Section A, that is, “Engagement” *(Engagement—fun, interesting, customizable, interactive—for example, sends alerts, messages, reminders, feedback, and enables sharing—and well targeted to audience*), and MARS item number 15 (*Quality of information: Is app content correct, well written, and relevant to the goal/topic of the app?*).

**Figure 2:**
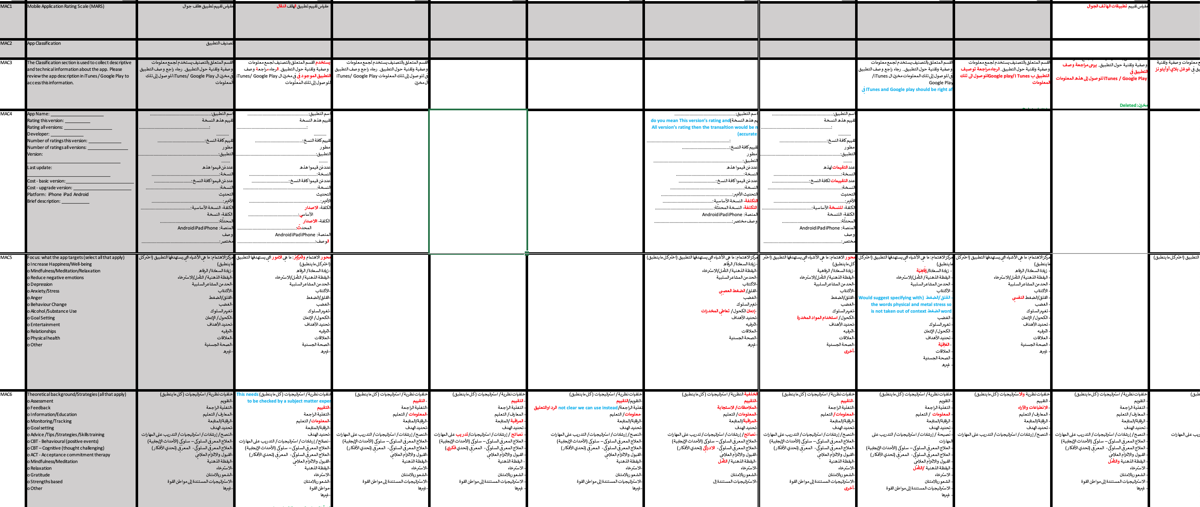
Comment tracking dashboard.

The research team created a matrix in Excel to track all comments and editing suggestions for each part of the translation. Each part was represented in rows, and the reviewers’ comments were organized in columns. This “comment tracking dashboard” (Figure 2) was used to visually compare and contrast the comments received from the reviewers, which were color coded to simplify the reviewing process.

**Figure 3:**
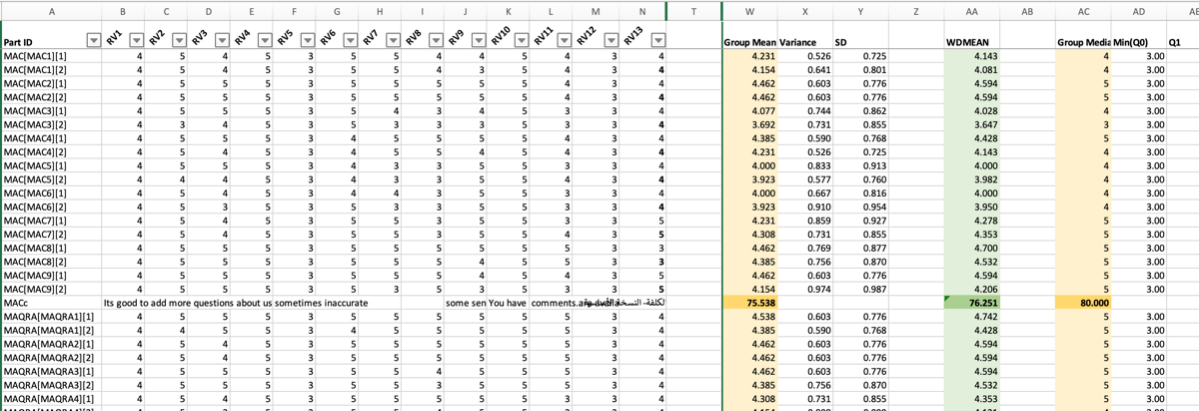
Interrater agreement dashboard.

We created a similar matrix in Excel to calculate the level of agreement among experts. The “Interrater agreement (IRA) dashboard” (Figure 3) was used to calculate variance, means, and medians used to establish interrater agreement (IRA) according to the three families of indices: James et al’s *r_WG(J)_* [47,48] (based on multiple null distributions [49]); Brown and Hauenstein’s *a_WG(J)_* [50]; and the adjusted average deviation index *A_DMJ(adj)_* [51]. IRA was established through pragmatic and theoretical cutoff points, such as for the *r_WG(J)_*: no agreement (<0.29), weak (0.30-0.49), moderate (0.50-0.69), strong (0.70-0.89), and very strong (>0.90) [52,53]; *a_WG(J)_*: not acceptable (<0.59), weak (0.60-0.69), moderate (0.70-0.79), and strong agreement (>0.80) [50]; and *A_DMJ(adj)_*: agreement above 0.80 [51].

The “IRA dashboard” showed that the 14 experts rated the translation as highly appropriate (mean 4.37, SD 0.16; range 4.00-4.69) and accurate (mean 4.26, SD 0.20; range 3.62-4.69). The level of agreement was acceptable for most items, except for the “Interactivity” item (the fourth item of the domain “Engagement”). The level of agreement for the accuracy was not acceptable only for two parts: the “Theoretical background/Strategies” and the “Technical aspects of app” in the “App Classification” section.

The research team also compiled a Word document including all editing suggestions and comments and printed out the Excel “comment matrix” to easily visualize the suggestions. The research team met and discussed each comment, spending more than 8 hours reviewing the editing suggestions for each part of the MARS tool. The most debated parts were those including technical terms such as “goal setting” and “mindfulness” or “wellness,” which did not find an established equivalent term in Arabic. Notable changes from the original MARS included the removal of context-specific references that were not relevant to the Arab world, such as research funding sources provided in MARS item number 18 (ie, “Australian Research Council and National Health and Medical Research Council”). Minor editing was done in the response options for item number 2 of “Subjective Quality” (“How many times do you think you would use this app in the next 12 months if it were relevant to you?”): the anchor texts were changed to 11-50 to avoid overlap with the third option choice (3-10).

After the revisions were completed, the research team shared the edited Word document on Google Docs with the same pool of reviewers who participated in the first round, who were invited to comment by email. After 12 days, 5 experts provided 107 additional editing suggestions. For the second round, the research team did not collect quantitative measures to reduce the burden on the reviewers, as most of the editing work had already been done. The research team met once again to address (accept or reject) all comments and finalized the document.

The final version of the document was sent to a second professional translator, who was not involved in the process and had not seen the original MARS tool. The developer of the MARS approved the back-translation of the MARS-Ar. This document was used in the validation study (further described below).

During the validation phase, one of the reviewers suggested some minor edits in the description of the “App Quality Ratings” part, in the description of the “Engagement” section, in the definition of “Target group” (item 5), in the description of the “Functionality” section, and in the items “Gestural design” (item 9) and “Graphics” (item 11). The research team approved the changes by circular vote. The final version of the MARS was then resent to the back-translator for verification. The final version of the MARS-Ar is available in Multimedia Appendix 1.

### Phases 3 and 4: Piloting and Validation

#### App Selection Process

**Figure 4:**
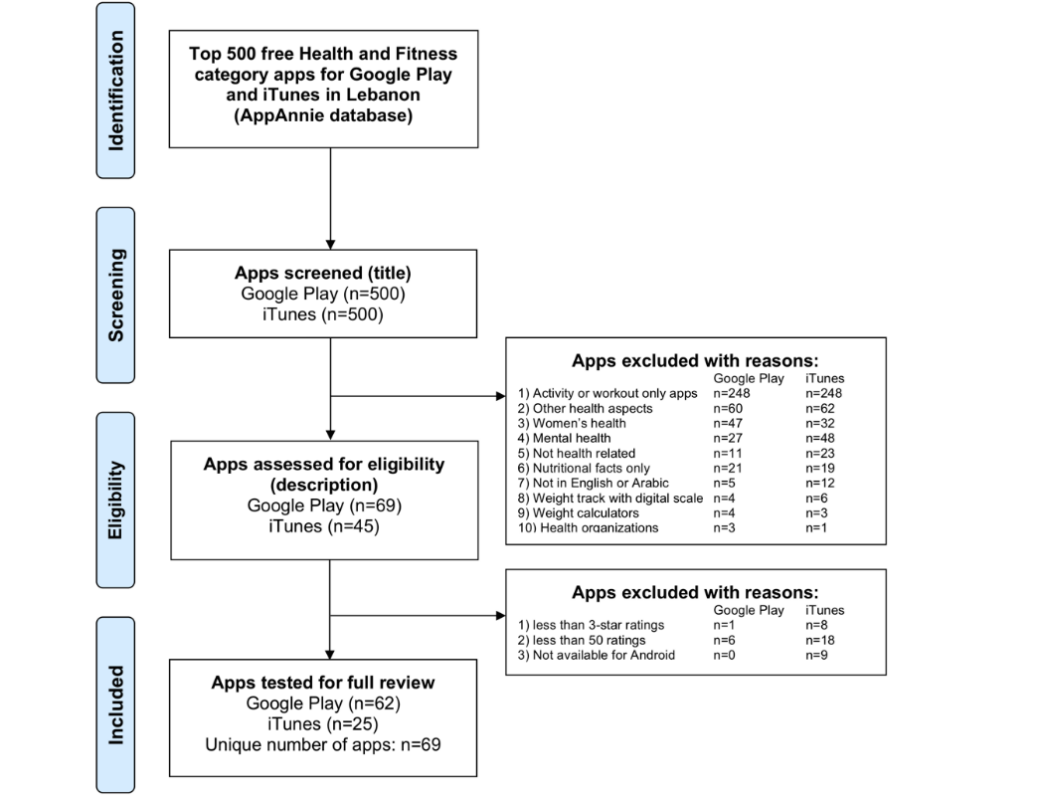
App selection process.

The research team identified the set of apps to be used in the piloting and validation phases of the study using the AppAnnie database (appannie.com), which provides updated rankings and mobile market data for both Android and iOS stores, under the section “App Store Rankings,” available after registering for free. On July 31, 2019, one researcher (MB) navigated the “Top Charts” section of the database, under the Google Play store, and filtered the country (Lebanon) and category (Health and Fitness) and selected the tab “Free” apps, extracting the titles and links to AppAnnie pages of 500 apps. These apps are listed under “free,” but in most cases, they operate under the “freemium” concept, with subscription fees used to remove ads and unlock complete features [54]. The researcher repeated the same procedure for the iOS store, as the apps’ rankings are quite different from the Google Play store, resulting in a second list of 500 apps. Links to AppAnnie’s webpages and titles of each app were imported in an Excel spreadsheet, to be screened for inclusion. The same researcher screened the lists and excluded irrelevant apps; a second researcher (NA) verified the selection. Any disagreement was discussed until consensus was reached. Of the total 1000 apps in both the Google Play store (Android) and the App Store (iOS), 431 and 455 apps were respectively excluded as they were not relevant (reasons for the exclusion are provided in the flowchart in Figure 4).

For the remaining 69 and 45 apps, the researchers extracted the following information from the AppAnnie’s database: ranking in the Health and Fitness category of the respective store (Google Play or App Store), number of ratings, average 5-star rating, date of first release, date of last update, number of installs category, and price (for monthly subscription or yearly subscription). The dates of the first release and last update were used to calculate the “app age” in years.

On the basis of the number of ratings and average rating, 7 and 20 apps were excluded from Google Play and App Store lists, respectively, as they did not receive at least three stars or were not rated by at least 50 people. The researchers created a combined database of 78 unique apps that were available from either Google Play or App Store lists. Of these, nine apps were excluded as they were available only on the App Store list. The resulting 69 apps were used to validate the MARS-Ar tool, as reviewers owned only Android phones. Although there might be slight differences in the apps across iOS and Android operating systems, we have already established that these differences are not substantial [38].

The research team decided that the number of apps was sufficient to have reasonable empirical assurance and reliability, based on the intraclass correlation coefficients (ICCs), as reported in the source study [31], used in the Italian translation study [40], and on the basis of formulas described in the study by Zou [55]. For the Italian translation, Domnich et al [40] calculated a minimum sample size of 41 apps for two raters to achieve an assurance probability of 0.15 and an empirical assurance of 90%.

#### Rater Training

Two researchers (NA and TA), fluent in both Arabic and English and with a background in pharmacy, public health, and nutrition, completed independent evaluations of the selected apps. One of the two researchers was based in Jordan and was familiar with the MARS, as the researcher had previously used it. The second researcher was based in Lebanon. Both researchers were instructed to view the “MARS training video” in English (about 37 min, available on YouTube upon request from the author of the MARS). Thereafter, they were instructed to download each app on their phones (F1 Plus x9009 and Samsung S7 Edge, both with Android 5.1) and use them for at least 10 min, reporting any incompatibility issues, if they arose. Once the app was thoroughly tested, they individually and independently completed a Web-based form containing the MARS-Ar, available on LimeSurvey. After they completed the review of the apps in Arabic, they received a link to complete a form containing the original MARS in English, to establish a minimum criterion of validity with a validated “gold standard” instrument. The reviewers did not have access to the information related to the apps so that users’ ratings or reviews could not influence their evaluations.

#### Piloting and Evaluation

The 2 raters completed a calibration exercise using the first 10 apps in the list to ensure that both understood the meaning of all terminology correctly and that they could carefully review and discuss any points of difference in their ratings. We calculated interrater reliability using ICCs, based on a two-way mixed effect model in which people effects are random and measures effects are fixed, based on the example of previous MARS translation studies [40,42]. Reliability was interpreted as excellent (ICCs≥0.90), good (ICCs: 0.76-0.89), moderate (ICCs: 0.51-0.75), and poor (ICCs≤0.50). The ICC based on the ratings of the first 10 apps (23 items×10=230 decisions per rater) was moderate (ICC=0.714, 95% CI 0.619-0.785). The two reviewers met with the first author to discuss every rating that varied by 2 points or more. During the meeting, both raters aligned their rating approaches and confirmed their correct understanding of all MARS-Ar terminology. It was deemed that no further amendments to the scale were necessary. Finally, the two raters independently revised their responses and completed the evaluation of the remaining 59 apps on the list.

#### Analyses: Reliability and Internal Consistency

To verify whether the two raters provided comparable results among all the tested apps so that ratings could be aggregated, we assessed interrater reliability through ICCs, as described above. Once interrater reliability was ascertained, the individual ratings for each item of the MARS-Ar and original MARS were averaged. The resulting items were used to calculate the respective subdomain scales of engagement, functionality, aesthetics, information quality, and subjective quality. A total app quality score was calculated as the average of engagement, functionality, aesthetics, and information quality.

We also assessed internal consistency as a measure of scale reliability for the items pertaining to the same subdomain of the MARS, as reported in the original MARS study [31]. We used Cronbach alpha indices, interpreted as excellent (≥.90), good (.80-.89), acceptable (.70-.79), questionable (.60-.69), poor (.50-.59), and unacceptable (<.50).

As an indicator of validity, we used Pearson correlations between each subdomain score of the MARS-Ar and the MARS equivalent. In addition, we correlated the total MARS-Ar score, the total subjective quality score, and the subjective quality item number 4 (5-star rating) with the 5-star ratings from the app store to understand the extent to which reviewers’ opinions about app quality were aligned with the users’ opinions. A cutoff point of *r*>0.80 was deemed a sufficient indication of the validity of the MARS-Ar instrument.

All statistical tests were conducted using SPSS v21 [56] for macOS (Apple Inc, Cupertino, California).

## Results

### Evaluated Apps

The two reviewers completed the evaluation of 67 out of 69 selected apps, using MARS-Ar, and 66 apps, using the MARS English version. One app was incompatible with both test devices, and 2 apps were not working on one of the two devices used. Another app became unavailable for one device, as it was removed from the Google Play store when one of the reviewers completed the MARS-English form*.* The dataset of the tested apps, with statistics about their ranking, ratings, and age (since their first development), is available in Multimedia Appendix 2 (Excel file).

### Interrater Reliability

The ICC based on the ratings for the full set of apps used in the MARS-Ar evaluation (23×67=1541 decisions per rater) was good (ICC=0.836, 95% CI 0.817-0.853). Similarly, the ICC for the English version (23×66=1518 decisions per rater) was also good (ICC=0.838, 95% CI 0.819-0.855).

### Internal Consistency

Table 1 shows the overall descriptive statistics for both MARS-Ar and MARS English. All domains of MARS-Ar and original MARS showed good internal consistency. For MARS-Ar, internal consistency was good for engagement (Cronbach alpha=.96) and aesthetics (alpha=.94), good for information quality (alpha=.81), and acceptable for functionality (alpha=.71). Similar indices were also reported for the original MARS.

Overall, the tested set of weight management apps had high functionality and aesthetic scores but low engagement, information quality, and subjective quality scores.

**Table 1:** Summary of Mobile App Rating Scale in Arabic and Mobile App Rating Scale-English items and subdomains means, SDs, and Cronbach alpha coefficients.

Mobile App Rating Scale domains and subdomains		Mobile App Rating Scale in Arabic		Mobile App Rating Scale in English	
		Mean (SD)	Alpha	Mean (SD)	Alpha
**Engagement**		2.94 (0.99)	.95	3.12 (0.93)	.95
	A1: Entertainment	2.69 (1.01)		2.78 (0.93)	
	A2: Interest	2.87 (1.15)		3.21 (1.05)	
	A3: Customization	2.69 (1.29)		2.86 (1.24)	
	A4: Interactivity	2.66 (1.23)		2.85 (1.14)	
	A5: Target group	3.78 (0.62)		3.89 (0.64)	
**Functionality**		4.11 (0.38)	.72	4.12 (0.32)	.73
	B1: Performance	3.91 (0.71)		4.00 (0.53)	
	B2: Ease of use	4.18 (0.37)		4.20 (0.30)	
	B3: Navigation	4.22 (0.42)		4.10 (0.44)	
	B4: Gestural design	4.13 (0.49)		4.17 (0.42)	
**Aesthetics**		3.14 (0.87)	.94	3.16 (0.72)	.96
	C1: Layout	3.65 (0.74)		3.55 (0.71)	
	C2: Graphics	3.00 (1.00)		2.98 (0.76)	
	C3: Visual appeal	2.78 (1.00)		2.95 (0.78)	
**Information quality**		2.53 (0.73)	.81	2.59 (0.68)	.82
	D1: Accuracy of app description	3.77 (0.64)		3.95 (0.53)	
	D2: Goals	3.29 (0.99)		3.30 (0.86)	
	D3: Quality of information	3.10 (0.90)		3.13 (0.75)	
	D4: Quantity of information	2.51 (0.98)		2.80 (0.77)	
	D5: Visual information	2.81 (1.86)		2.64 (1.86)	
	D6: Credibility	1.99 (0.63)		1.90 (0.48)	
	D7: Evidence base	0.27 (0.96)		0.42 (0.98)	
**Subjective quality**		2.21 (0.97)	.97	2.09 (0.79)	.95
	SQ1: Would you recommend it?	2.34 (1.07)		2.11 (0.85)	
	SQ2: How many times would you use it?	1.96 (1.03)		1.84 (0.87)	
	SQ3: Would you pay for it?	1.75 (0.91)		1.67 (0.73)	
	SQ4: 5-star rating	2.81 (1.07)		2.74 (0.90)	
Total app quality		3.18 (0.69)	—^a^	3.24 (0.61)	—

^a^Chronbach alpha for total app quality is not computed.

### Mobile App Rating Scale in Arabic Validity

The correlations between MARS-Ar and original MARS and among each domain are presented diagonally in Table 2. The correlations among the domains of engagement, functionality, aesthetics, information quality, total app quality, and subjective quality are presented in the upper off-diagonal (for Arabic) and lower off-diagonal (for English).

The correlations between MARS-Ar and MARS-English were all significant at *P*<.001. The lowest was found in the domain of functionality (*r*=0.685), followed by aesthetics (*r*=0.827), information quality (*r*=0.854), engagement (*r*=0.894), and total app quality (*r*=0.897). Subjective quality scores and the item number 4 (5-star rating) were also highly correlated (*r*=0.820).

The 5-star rating from the app stores was not significantly associated with any app quality subdomain, total app quality, subjective quality, or MARS 5-star rating, neither in the Arabic nor in the English version.

**Table 2:** Correlations between Mobile App Rating Scale in Arabic and Mobile App Rating Scale-English domains and total app quality.

Mobile App Rating Scale in Arabic	Mobile App Rating Scale in English								5-star rating
	A	B	C	D	App quality	E	E4		
Engagement (A)	0.89^a,b^	0.64^a,c^	0.90^a,c^	0.92^a,c^	0.97^a,c^	0.90^a,c^	0.88^a,c^	−0.04^c^	
Functionality (B)	0.61^a,d^	0.69^a,b^	0.70^a,c^	0.61^a,c^	0.75^a,c^	0.70^a,c^	0.48^a,c^	−0.03^c^	
Aesthetics (C)	0.89^a,d^	0.68^a,d^	0.83^a,b^	0.86^a,c^	0.96^a,c^	0.91^a,c^	0.78^a,c^	0.03^c^	
Information Quality (D)	0.92^a,d^	0.55^a,d^	0.81^a,d^	0.85^a,b^	0.95^a,c^	0.84^a,c^	0.80^a,c^	−0.14^c^	
App quality score (average A-D)	0.96^a,d^	0.72^a,d^	0.95^a,d^	0.93^a,d^	0.90^a,b^	0.92^a,c^	0.84^a,c^	−0.05^c^	
Subjective quality score (E)	0.88^a,d^	0.67^a,d^	0.82^a,d^	0.87^a,d^	0.90^a,d^	0.82^a,b^	0.78^a,c^	−0.07^c^	
Subjective quality item number 4: 5-star rating (E4)	0.83^a,d^	0.62^a,d^	0.80^a,d^	0.78^a,d^	0.85^a,d^	0.83^a,d^	0.82^a,b^	0.00^c^	
5-star rating (app stores)	−0.05^d^	0.04^d^	0.03^d^	−0.11^d^	−0.04^d^	−0.07^d^	−0.09^d^	1.00^b^	

^a^*P*<.001.

^b^The diagonal shows the correlations between the same constructs of the MARS English and Arabic.

^c^In the upper diagonal section of the table: correlations among Mobile App Rating Scale subdomains, total app quality, and subjective quality (Mobile App Rating Scale in Arabic).

^d^In the lower diagonal section of the table: correlations among Mobile App Rating Scale subdomains (English).

## Discussion

### Principal Findings

This study aimed at translating and adapting MARS-Ar and at validating this scale with a set of popular health and fitness apps promoting weight management. The translation process demonstrated the importance of involving expert translators with interest and experience in translating technology-related documents. English-Arabic translation is not an easy task, as the language has many different regional varieties that make it difficult to find words that are common to the Modern Standard Arabic (MSA) dictionary [57]. In the literature related to English-Arabic translations, it is common to find reports of challenges related to the nonequivalence of words and sentence structures between the two languages [58], which occurs when translating colloquial or legal documents [59]. It was also important to involve experts from different countries of the Arab world, who provided valuable feedback and suggestions for improvement, as there are significant differences between the MSA and the many regional varieties (eg, Levantine Arabic vs Saudi or Gulf-countries or the Maghreb), with a plethora of dialects and different spoken expressions [60,61]. We found it challenging to find accurate translations of some technical terms and concepts referring to MARS domains, such as “Interactivity” or “Engagement,” which was also the case for some general terms, such as “goal setting” and “mindfulness” or “wellness,” usually used in disciplines such as Psychology and Health Sciences, usually taught in English; hence, the translations in Arabic were not easy to find.

After two rounds of review and additional feedback collected during the validation phase, we are confident to have a good instrument that Arabic-speaking researchers and experts can use to evaluate app quality in their native language. It is essential that Arabic-speaking researchers or professionals interested in evaluating apps establish a good and acceptable interrater reliability level before evaluating the full set of apps (ie, ICC above 0.70), as recommended in the MARS-German validation study [42]. A training video, similar to the one for MARS, will be developed so that the interpretation of terminology across researchers of different backgrounds and countries can be kept consistent.

This study’s results show that MARS-Ar is a reliable and valid instrument that trained “experts” can use to assess the quality of health apps. From a quantitative standpoint, there were no substantial differences in the reliabilities between the MARS-Ar and the original MARS in English. All MARS-Ar subdomains and individual quality items achieved appropriate internal consistency, comparable with the source study [31] and comparable with those reported in Italian [40] and German [42] validation studies. Similar to the German and Italian validation studies, the correlations between each subdomain of MARS-Ar and the original counterparts were also significant and extensive in size, indicating that the instrument tends to be valid.

In this study, we also found that the app quality ratings, according to experts, are not associated with the 5-star ratings reported in the app stores. These findings are consistent with another similar app review comparing expert ratings with the app stores [38] and with the MARS-German validation study [42]. App quality appears to be a complicated concept, which goes beyond a 5-star rating, as used in app stores. These ratings are not necessarily linked to the quality of health apps [62], as they can be inflated by developers [63]. With a sizeable and significant turnover of health apps [23], end users tend to rely on quick and available information to determine whether an app is worth downloading. MARS, as it is short and easy to understand and apply, could become the standard for app quality evaluation and provide researchers and end users with comparable dimensions across app domains.

With more versions of MARS available—Italian [40], Spanish [41], German [42], and now Arabic—it will be possible to complete cross-cultural app evaluations and develop a joint research database of app evaluations, which could be made accessible to end users. Future studies should aim at involving end users to compare the ratings, for example, using the ratings between the uMARS and MARS versions.

The proposed project has a multifold impact. First, it provides Arab-speaking researchers and public health professionals, operating in the MENA region and elsewhere, with a culturally adapted and validated tool that could be used for developing new and evaluating existing apps. Second, this study will test whether MARS-Ar and uMARS in Arabic could be used to reliably evaluate the quality of apps for the prevention and treatment of obesity and related NCDs. Third, it can fulfill the needs of millions of people living in the region, who might be interested in knowing which apps could be trusted to prevent or better manage these conditions. Once the validation of the tool has been established, the researchers will maintain a database of app evaluations, thereby increasing the applicability and comparability of the results across multiple apps targeting the same public health issues.

### Limitations

Despite its strengths, this study has some limitations to be acknowledged. First, the validity of the MARS-Ar instrument was established by comparing the scales in Arabic with their equivalents in the original MARS instrument, which the same raters completed in English. Future studies may compare MARS to other instruments of app quality [23,30], even though they might not be equivalent. We tested MARS-Ar with a set of apps for weight management; therefore, future studies need to test whether this instrument could also apply to health apps of different domains.

### Conclusions

This study shows that MARS-Ar is a valid instrument, which can be used to assess app quality among trained Arabic-speaking users of health and fitness apps. Researchers and public health professionals in the Arab world can use the overall MARS score and its subscales to reliably evaluate the quality of weight management apps. Further studies are needed to test the instrument on health apps focusing on different health domains that are covered in health and fitness apps, such as mindfulness/anxiety prevention or sexual and reproductive health.

## Appendix

Multimedia Appendix 1 Arabic version of the Mobile App Rating Scale.

Multimedia Appendix 2 Excel spreadsheet including the apps used in the validation study.

## Abbreviations

ICC: intraclass correlation coefficient
IRA: interrater agreement
MARS: Mobile App Rating Scale
MARS-Ar: Mobile App Rating Scale in Arabic
MENA: Middle East and North Africa
mHealth: mobile health
MSA: Modern Standard Arabic
NCD: noncommunicable disease
uMARS: user version of the Mobile App Rating Scale

## References

[1] (2013). World Health Organization.

[2] Rahim HF, Sibai A, Khader Y, Hwalla N, Fadhil I, Alsiyabi H, Mataria A, Mendis S, Mokdad AH, Husseini A (2014). Non-communicable diseases in the Arab world. Lancet.

[3] Musaiger A (2011). Overweight and obesity in eastern mediterranean region: prevalence and possible causes. J Obes.

[4] Musaiger AO, Al-Mannai M, Al-Lalla O, Saghir S, Halahleh I, Benhamed MM, Kalam F, Ali EY (2013). Obesity among adolescents in five Arab countries; relative to gender and age. Nutr Hosp.

[5] Badran M, Laher I (2012). Type II Diabetes Mellitus in Arabic-Speaking countries. Int J Endocrinol.

[6] Hyder AA, Wosu AC, Gibson DG, Labrique AB, Ali J, Pariyo GW (2017). Noncommunicable disease risk factors and mobile phones: a proposed research agenda. J Med Internet Res.

[7] The Lancet (2017). Does mobile health matter?. Lancet.

[8] Royston G, Hagar C, Long L, McMahon D, Pakenham-Walsh N, Wadhwani N, mHIFA Working Group (Mobile Healthcare Information For All) (2015). Mobile health-care information for all: a global challenge. Lancet Glob Health.

[9] International Telecommunication Union, World Health Organization (2016). Be He@lthy, Be Mobile. Annual Report 2016.

[10] (2018). Statista.

[11] Semper HM, Povey R, Clark-Carter D (2016). A systematic review of the effectiveness of smartphone applications that encourage dietary self-regulatory strategies for weight loss in overweight and obese adults. Obes Rev.

[12] Aguilar-Martínez A, Solé-Sedeño JM, Mancebo-Moreno G, Medina FX, Carreras-Collado R, Saigí-Rubió F (2014). Use of mobile phones as a tool for weight loss: a systematic review. J Telemed Telecare.

[13] Bardus M, Smith JR, Samaha L, Abraham C (2015). Mobile phone and web 2.0 technologies for weight management: a systematic scoping review. J Med Internet Res.

[14] Quelly SB, Norris AE, DiPietro JL (2016). Impact of mobile apps to combat obesity in children and adolescents: a systematic literature review. J Spec Pediatr Nurs.

[15] Riaz S, Sykes C (2015). Are smartphone health applications effective in modifying obesity and smoking behaviours? A systematic review. Health Technol.

[16] Mateo GF, Granado-Font E, Ferré-Grau C, Montaña-Carreras X (2015). Mobile phone apps to promote weight loss and increase physical activity: a systematic review and meta-analysis. J Med Internet Res.

[17] Bardus M, Smith JR, Samaha L, Abraham C (2016). Mobile and Web 2.0 interventions for weight management: an overview of review evidence and its methodological quality. Eur J Public Health.

[18] Schippers M, Adam PC, Smolenski DJ, Wong HT, de Wit JB (2017). A meta-analysis of overall effects of weight loss interventions delivered via mobile phones and effect size differences according to delivery mode, personal contact, and intervention intensity and duration. Obes Rev.

[19] (2017). Research 2 Guidance.

[20] (2017). Research and Markets.

[21] Mikulich M (2019). Statista.

[22] Statista.

[23] Larsen ME, Nicholas J, Christensen H (2016). Quantifying app store dynamics: longitudinal tracking of mental health apps. JMIR Mhealth Uhealth.

[24] Becker S, Miron-Shatz T, Schumacher N, Krocza J, Diamantidis C, Albrecht U (2014). mHealth 2.0: experiences, possibilities, and perspectives. JMIR Mhealth Uhealth.

[25] Alnasser AA, Alkhalifa AS, Sathiaseelan A, Marais D (2015). What overweight women want from a weight loss app: a qualitative study on arabic women. JMIR Mhealth Uhealth.

[26] Azar KM, Lesser LI, Laing BY, Stephens J, Aurora MS, Burke LE, Palaniappan LP (2013). Mobile applications for weight management: theory-based content analysis. Am J Prev Med.

[27] Nikolaou CK, Lean MEJ (2017). Mobile applications for obesity and weight management: current market characteristics. Int J Obes (Lond).

[28] Pagoto S, Schneider K, Jojic M, DeBiasse M, Mann D (2013). Evidence-based strategies in weight-loss mobile apps. Am J Prev Med.

[29] BinDhim NF, Hawkey A, Trevena L (2015). A systematic review of quality assessment methods for smartphone health apps. Telemed J E Health.

[30] Grundy QH, Wang Z, Bero LA (2016). Challenges in assessing mobile health app quality: a systematic review of prevalent and innovative methods. Am J Prev Med.

[31] Stoyanov SR, Hides L, Kavanagh DJ, Zelenko O, Tjondronegoro D, Mani M (2015). Mobile app rating scale: a new tool for assessing the quality of health mobile apps. JMIR Mhealth Uhealth.

[32] Wilson H, Stoyanov SR, Gandabhai S, Baldwin A (2016). The quality and accuracy of mobile apps to prevent driving after drinking alcohol. JMIR Mhealth Uhealth.

[33] Furlong LM, Morris ME, Erickson S, Serry TA (2016). Quality of mobile phone and tablet mobile apps for speech sound disorders: protocol for an evidence-based appraisal. JMIR Res Protoc.

[34] Creber RM, Maurer MS, Reading M, Hiraldo G, Hickey KT, Iribarren S (2016). Review and analysis of existing mobile phone apps to support heart failure symptom monitoring and self-care management using the Mobile Application Rating Scale (MARS). JMIR Mhealth Uhealth.

[35] Mani M, Kavanagh DJ, Hides L, Stoyanov SR (2015). Review and evaluation of mindfulness-based iPhone apps. JMIR Mhealth Uhealth.

[36] Zini F, Reinstadler M, Ricci F, Giokas K, Bokor L, Hopfgartner F (2017). Increasing quality of life awareness with life-logging. eHealth 360°.

[37] Patel R, Sulzberger L, Li G, Mair J, Morley H, Shing MN, O'Leary C, Prakash A, Robilliard N, Rutherford M, Sharpe C, Shie C, Sritharan L, Turnbull J, Whyte I, Yu H, Cleghorn C, Leung W, Wilson N (2015). Smartphone apps for weight loss and smoking cessation: quality ranking of 120 apps. N Z Med J.

[38] Bardus M, van Beurden SB, Smith JR, Abraham C (2016). A review and content analysis of engagement, functionality, aesthetics, information quality, and change techniques in the most popular commercial apps for weight management. Int J Behav Nutr Phys Act.

[39] Stoyanov SR, Hides L, Kavanagh DJ, Wilson H (2016). Development and validation of the user version of the mobile application rating scale (uMARS). JMIR Mhealth Uhealth.

[40] Domnich A, Arata L, Amicizia D, Signori A, Patrick B, Stoyanov S, Hides L, Gasparini R, Panatto D (2016). Development and validation of the Italian version of the Mobile Application Rating Scale and its generalisability to apps targeting primary prevention. BMC Med Inform Decis Mak.

[41] Payo RM, Álvarez MM, Díaz MB, Izquierdo MC, Stoyanov S, Suárez EL (2019). Spanish adaptation and validation of the Mobile Application Rating Scale questionnaire. Int J Med Inform.

[42] Messner E, Terhorst Y, Barke A, Baumeister H, Stoyanov S, Hides L, Kavanagh D, Pryss R, Sander L, Probst T (2019). Development and validation of the German version of the Mobile Application Rating Scale (MARS-G). JMIR Preprints.

[43] EF Education First.

[44] Herdman M, Fox-Rushby J, Badia X (1998). A model of equivalence in the cultural adaptation of HRQoL instruments: the universalist approach. Qual Life Res.

[45] Epstein J, Santo RM, Guillemin F (2015). A review of guidelines for cross-cultural adaptation of questionnaires could not bring out a consensus. J Clin Epidemiol.

[46] LimeSurvey.

[47] James LR, Demaree RG, Wolf G (1984). Estimating within-group interrater reliability with and without response bias. J Appl Psychol.

[48] James LR, Demaree RG, Wolf G (1993). rwg: An assessment of within-group interrater agreement. J Appl Psychol.

[49] Biemann T, Cole MS, Voelpel S (2012). Within-group agreement: On the use (and misuse) of rWG and rWG(J) in leadership research and some best practice guidelines. Leadersh Q.

[50] Brown RD, Hauenstein NM (2005). Interrater agreement reconsidered: an alternative to the rwg indices. Organ Res Methods.

[51] Lohse-Bossenz H, Kunina-Habenicht O, Kunter M (2014). Estimating within-group agreement in small groups: a proposed adjustment for the average deviation index. Eur J Work Organ Psychol.

[52] LeBreton JM, Senter JL (2008). Answers to 20 questions about interrater reliability and interrater agreement. Organ Res Method.

[53] O'Neill TA (2017). An overview of interrater agreement on Likert scales for researchers and practitioners. Front Psychol.

[54] Kosner AW (2015). Forbes.

[55] Zou GY (2012). Sample size formulas for estimating intraclass correlation coefficients with precision and assurance. Stat Med.

[56] IBM.

[57] Benzehra R (2012). Issues and challenges for a modern English-Arabic dictionary. Dictionaries.

[58] Chahrour O (2017). Translation Journal.

[59] Zidan AA (2015). A Linguistic Analysis of Some Problems of Arabic-English Translation of Legal Texts, with Special Reference to Contracts.

[60] Huddleston G (2017). Language Connect.

[61] Huddleston G (2017). Language Connect.

[62] Plante T, O'Kelly AC, Macfarlane Z, Urrea B, Appel L, Miller III ER, Blumenthal R, Martin S (2018). Trends in user ratings and reviews of a popular yet inaccurate blood pressure-measuring smartphone app. J Am Med Inform Assoc.

[63] Hill S (2018). Digital Trends.

