# Supplementary material for: The Arabic Version of the Mobile App Rating Scale: Development and Validation Study
Source: JMIR Mhealth Uhealth. 2020 Mar 3;8(3):e16956. doi: 10.2196/16956 (PMC7078658; doi:10.2196/16956)
Supplement: Multimedia Appendix 1 [file mhealth_v8i3e16956_app1.docx]

# **مقياس تقييم تطبيق الهاتف الجوال**

**تصنيف التطبيق**

**يالقسم المتعلق بالتصنيف لجمع معلومات وصفية وتقنية حول التطبيق. رجاء راجع وصف التطبيق في للوصول إلى تلك المعلومات iTunes/ Google Play**

**اسم التطبيق:................................................**

**تقييم هذه النسخة :.................................................................**

**تقييم كافة النسخ:.....................................**

**مطور التطبيق:......................................................................**

**عدد التقيمات لهذه النسخة:.....................................................**

**عدد التقييمات لكافة النسخ:.............................**

**النسخة:..........................................................**

**التحديث الأخير:........................................................**

**الكلفة- النسخة الأساسية:.......................................**

**الكلفة- النسخة المحدثّة:..............................................**

**المنصة: iPhone iPad Android**

**وصف مختصر:.............................................................**

**محور التطبيق: ما يستهدفه التطبيق (اختر كل ما ينطبق)**

- **زيادة السعادة/ الرفاه**
- **اليقظة الذهنية/ التأمل/الاسترخاء**
- **الحد من المشاعر السلبية**
- **الاكتئاب**
- **القلق/الضغط النفسي**
- **الغضب**
- **تغيير السلوك**
- **الكحول/ إستخدام المواد المسببة للإدمان**

**تحديد الأهداف**

- **الترفيه**
- **العلاقات**
- **الصحة الجسدية**
- **غيره**

**الخلفية النظرية / الإستراتيجيات (كل ما ينطبق)**

- التقييم
- **التغذية الراجعة**
- **المعلومات / التعليم**
- **المراقبة /المتابعة**
- **, تحديدالهدف**
- **النصح/ الإرشادات / الاستراتيجيات/ التدريب على المهارات**
- **العلاج المعرفي السلوكي – سلوكي (الأحداث الإيجابية)**
- **العلاج المعرفي السلوكي- المعرفي (تحدي الأفكار)**
- **القبول والالتزام العلاجي**
- **اليقظة الذهنية /التأمل**
- **الاسترخاء**
- **الشعور بالامتنان**
- **النهج المبني على نقاط القوة**
- **غيرها**
- **انتسابات (مطورو التطبيق)**
- **غير معروف**
- **تجارية**
- **حكومية**
- منظمات غير حكومية
- **جامعية**

**الفئة العمرية (كل ما ينطبق)**

- **الأطفال ( دون 12 سنة)**
- **المراهقون (13-17)**
- **الشباب (18-25)**
- **البالغون**
- **كل الفئات**

الجوانب التقنية للتطبيق (كل ما ينطبق)

- يسمح بالمشاركة (Facebook, Twitter، إلخ )
- يمتلك التطبيق مجتمعاً
- يسمح بحماية عبر كلمة مرور
- يتطلب تسجيل الدخول
- يرسل تذكير
- بحاجة إلى الاتصال بشبكة الإنترنت للتشغيل

**تقييم نوعية التطبيق**

يقيس مقياس التقييم جودة التطبيق بحسب أربعة أبعاد. كل بعد يتم تقييمه بحسب مقياس مكون من خمس نقاط "1 غير ملائم" إلى 5 ممتاز". ضع دائرة حول الرقم الذي يمثل بدقة جودة مكون التطبيق الذي تقوم بتقييمه. الرجاء إستخدام التوصيفات المقدمة لكل إجابة.

**القسم** A

**الجذب - مسلي، مثير للإهتمام، قابل للتخصيص، متفاعل (مثال: يرسل تنبيهات، رسائل، تذكيرات ، تغذية راجعة، يفسح المجال للمشاركة) مناسب للمستخدم المستهدف**

الترفيه: هل التطبيق مسل/ ترفيهي عند الاستخدام ؟ هل يستخدم أي استراتيجيات لزيادة الجذب عبر الترفيه (من خلال اللعب التلعيب(

1. **ممل، ليس مسل بتاتا**
2. **ممل في معظم الأحيان**
3. **مقبول ، مسل للمستخدم ولكن لفترة وجيزة (أقل من 5 دقائق )**
4. **مسل وترفيهي بشكل متوسط، يسلي المستخدم لبعض الوقت ( 5-10 دقائق )**
5. **مسل وترفيهي إلى حد كبير، ويحفز الاستخدام المتكرر**

التشويق : هل التطبيق مشوق لدرجة تدفع إلى استخدامه؟ هل يستخدم أي استراتيجية لزيادة الجذب عبر عرض محتواه بطريقة مشوقة؟

**1 ليس مشوقاً بتاتا**

**2 غير مشوق في معظم الأحيان**

**3 مقبول ، ليس مشوقاً ولا غير مشوق؛ سيجعل المستخدم منغمسا لفترة محدودة ( أقل من 5 دقائق)**

1. **مشوق بدرجة متوسطة: سيجعل المستخدم منغمسا لبعض الوقت (5-10 دقائق)**

**5 مشوق جدا، سيجعل المستخدم منغمساً**

القابلية للتخصيص : هل يوفر/ يحتفظ بكافة الإعدادات/ التفضيلات المتعلقة بمزايا التطبيقات ( مثال: الصوت، المحتوى، الإخطارات، إلخ )؟

**1 لا يسمح بالتخصيص أو يتطلب إدخال الإعدادات في كل مرة**

**2 يسمح بالتخصيص ولكن بشكل غير كاف مما يحد من الوظائف**

**3 يسمح بتخصيص أساسي مما يجعله يعمل بشكل كاف**

**4 يسمح بعدة خيارات متعلقة بالتخصيص**

**5** يسمح بتخصيص كامل طبقا لتفضيلات الفرد **يحتفظ بالإعدادات**

التفاعلية : هل يسمح بإدخال مدخلات من قبل المستخدم، يقدم تغذية راجعة، يحتوي على تنبيهات (تذكير، المشاركة في الخيارات، تبليغات، إلخ) ؟ ملاحظة: هذه الوظائف تحتاج أن تكون قابلة للتخصيص بدون عناء كي تكون مثالية.

1. **لا خصائص تفاعلية و/أو لا استجابة للتفاعل المقدم من المستخدِم**
2. **التفاعلية ، التغذية الراجعة، أو خيارات لمدخلات المستخدم غير كافية مما يحد من وظائف التطبيق**
3. **خصائص تفاعلية أساسية لتأدية الوظائف بشكل كاف**
4. **يقدم خصائص تفاعلية متنوعة/ تغذية راجعة/ خيارات لمدخلات من قبل المستخدم**
5. **استجابة عالية جدا عبر خصائص تفاعلية / تغذية راجعة/ خيارات لمدخلات من قبل المستخدم**

**المجموعة المستهدفة: هل محتوى التطبيق (المعلومات البصرية، اللغة، التصميم) ملائم للمستخدم المستهدف؟**

- - 1. غير ملائم تماما/ غير واضح/ محيّر
    2. غالبا غير مناسب / غير واضح/ محيّر
    3. مقبول لكنه غير موجه للمجموعة المستهدفة. قد يكون غير مناسب / غير واضح/ محيّر
    4. موجه بشكل مناسب، مع وجود ملاحظات غيرمهمة
    5. موجه بشكل تام، لا ملاحظات

A . متوسط مقياس الجذب =

**القسم B**

**الوظيفية- أداء التطبيق، سهولة التعلم، التصفح ،** منطقية الانتقال **، التصميم الإيمائي للتطبيق**

الأداء: ما هي درجة الدقة/ السرعة التي تعمل فيها مواصفات (وظائف) التطبيق ومكوناته (الأزرار والقائمة)؟

1. **التطبيق معطل ؛ لا استجابة/ استجابة غير كافية/ استجابة غير دقيقة ( مثال: تعطل التطبيق/ أخطاء في التطبيق/ خصائص معطلة**
2. **بعض الوظائف تعمل، لكنها تتأخر أو تحوي مشاكل تقنية كبيرة**
3. **يعمل التطبيق بشكل عام. بعض المشاكل التقنية تحتاج إلى إصلاح/ بطيء أحيانا**
4. **يعمل في معظم الأحيان ولكن بوجود مشاكل بسيطة/ لا تذكر**
5. **استجابة ممتازة/ في الوقت المناسب؛ لا وجود لأخطاء في التطبيق/ يحوي مؤشر "للوقت المتبقي للتحميل"**

سهولة الاستخدام: ما مدى سهولة تعلم استخدام التطبيق؛ ما مدى وضوح قائمة الاختيار/ الأيقونات والتعليمات ؟

- 1. **لا تعليمات/التعليمات محدودة؛ علامات قائمة الاختيار/ الأيقونات محيرة؛ معقد**
  2. **صالح للاستخدام بعد وقت/ جهد كبيرين**
  3. **صالح للاستخدام بعد قضاء بعض الوقت/ الجهد**
  4. **يسهل تعلم استخدام التطبيق (أو يحوي تعليمات واضحة)**
  5. **القدرة على استخدام التطبيق مباشرة؛ بديهي؛ بسيط**

**التصفح**: هل الانتقال بين الشاشات منطقي/ دقيق/ مناسب / ومتواصل؛ هل تتوافر كافة روابط الشاشة الضرورية؟

- 1. أقسام التطبيق المختلفة غير مترابطة بشكل منطقي و**التصفح** يكون عشوائيا ومحيرا/ **التصفح** يكون صعبا
  2. **صالح للاستخدام بعد وقت / جهد كبيرين**
  3. **صالح للاستخدام بعد قضاء بعض الوقت/ الجهد**
  4. سهل الاستخدام **أو يفتقد إلى رابط غير مهم**
  5. **التنقل بين الشاشات منطقي، سهل، واضح وبديهي، او يوفر اختصارات**

**التصميم الإيمائي: هل التفاعل بواسطة ( الضغط، السحب الى اليمين او اليسار، الانتقال الى الاعلى او الاسفل) متناسق و بديهي عبر كل المكونات/الشاشات؟**

1. غير متناسق تماما/ محير
2. عادة ما يكون غير متناسق / محيرا
3. مقبول مع بعض العناصر غير المتناسقة / المحيرة
4. في معظم الأحيان متناسق / بديهي بوجود مشاكل محدودة
5. متناسق وبديهي على نحو كامل

**B**. متوسط مقياس الوظيفية =

**القسم C**

**الجماليات- التصميم الجرافيكي، الجاذبية البصرية بشكل عام، نظام الألوان ، الاتساق الأسلوبي**

**التصميم : هل تنسيق وحجم الأزرار/ الأيقونات/ قائمة الإعداد/ المحتوى على الشاشة مناسب أو قابل للتكبير أو التصغير إذا دعت الحاجة؟**

1. تصميم سيء جداً، مبعثر، بعض الخيارات يستحيل اختيارها/تحديد موقعها/ رؤيتها/ قراءتها –عرض التطبيق على شاشة الجهاز ليست في الوضع المثالي
2. تصميم سيء، عشوائي ، غير واضح، بعض الخيارات يصعب اختيارها/ تحديد موقعها/ رؤيتها/ قراءتها
3. مرضٍ، مشاكل محدودة في اختيار/ تحديد موقع/ رؤية/ قراءة العناصر أو مشاكل محدودة في حجم العرض على الشاشة
4. واضح في معظم الأحيان، قادر على اختيار/ تحديد موقع/ رؤية/ قراءة العناصر
5. احترافي، بسيط، واضح، مرتب، منظم بشكل منطقي، شاشة عرض الجهاز في وضعها المثالي. لكل مكون من مكونات التصميم هدف.

**الرسومات: ما مدى جودة/ وضوح الرسومات المستخدمة للأزرار/ الأيقونات/ قوائم الإعداد/ المحتوى؟**

1. الرسومات تبدو بدائية، تصميم بصري ضعيف جدا- غير متناسق، ذو أسلوب غير متناسق بشكل تام
2. جودة متدنية/ عدم وضوح **الرسومات** ؛ تصميم بصري ذو جودة متدنية- غير متناسق، ذو أسلوب غير متناسق
3. نوعية متوسطة للرسومات والتصميم البصري ( عادة هناك اتساق في الأسلوب )
4. جودة عالية/ وضوح عال للرسومات والتصميم البصري- تناسق في معظم الأحيان، اتساق أسلوبي
5. جودة عالية جدا/ وضوح كبير في الرسومات التصميم البصري- تناسق، اتساق أسلوبي على الدوام

**الجاذبية البصرية: إلى أي مدى يبدو التطبيق جذابا؟**

1. لا جاذبية بصرية، منظره غير حسن، تصميمه سيء، ألوان متضاربة/ غير متلائمة مع بعضها البعض
2. جاذبية بصرية محدودة- تصميمه سيء، استخدام سيء للألوان، ممل بصرياً
3. فيه بعض الجاذبية البصرية – ضمن المعدل، ليس جذاباً، وليس سيئاً
4. جاذبية بصرية عالية – رسومات مترابطة- مصمم باتساق وحرفية
5. كما في البند 4 بالإضافة لكونه جذابا جداً، يمكن تذكره ، بارز؛ استخدام الألوان يحسن من مواصفات التطبيق/ قائمة الإعداد

ج. النمتوسط مقياس الجماليات =.........................................................

**القسم D**

المعلومات - يحتوي على معلومات ذات جودة عالية (مثال، نصوص، تغذية راجعة، مقاييس، مراجع) ذات مصادر موثوقة. اختر غ/م في حال كان المكون غير ذي صلة

**الدقة في وصف التطبيق (في متجر التطبيقات ): هل يحتوي التطبيق على ما تم وصفه في متجر التطبيقات؟**

1 مضلِّل. لا يحتوي التطبيق على المكونات/ الوظائف التي تم وصفها. أو لا يوجد هناك أي وصف

1. غير دقيق. يحتوي التطبيق على مكونات/وظائف محدودة من التي تم وصفها
2. مقبول. يحتوي التطبيق على بعض المكونات/ الوظائف التي تم وصفها
3. دقيق. يحتوي التطبيق على معظم المكونات/ الوظائف التي تم وصفها
4. وصف دقيق جداً لمكونات/ وظائف التطبيق

**الأهداف: هل للتطبيق أهداف محددة، يمكن قياسها، ويمكن تحقيقها ( مذكورة في وصف متجر التطبيقات أو في التطبيق نفسه)؟**

غ/م الوصف لا يسرد الأهداف، أو أهداف التطبيق لا علاقة لها بهدف البحث (مثل استخدام لعبة للأغراض التعليمية)

1. لا فرصة لتحقيق الأهداف المعلنة
2. يذكر الوصف بعض الأهداف، لكن الفرصة محدودة لتحقيقها
3. مقبول . للتطبيق أهداف واضحة، والتي يمكن أن تكون قابلة للتحقيق
4. للتطبيق أهداف واضحة ومحددة يمكن قياسها و قابلة للتحقيق
5. للتطبيق أهداف محددة يمكن قياسها ويمكن تحقيقها وتكون قابلية تحقيقها مرجحة جدا.

**جودة المعلومات: هل محتوى التطبيق صحيح، مُصاغ بشكل جيد، مرتبط بهدف/ موضوع التطبيق؟**

غ/م لا تتوافر معلومات ضمن التطبيق

1. غير ذات صلة / غير مناسبة / مفككة / خاطئة
2. ضعيفة. بالكاد تكون ذا صلة/ مناسبة/ متماسكة/ وتبدو صحيحة
3. ذات صلة/ مناسبة/ متماسكة بدرجة متوسطة/وتبدو صحيحة
4. ذات صلة/ مناسبة/ متماسكة/ صحيحة
5. ذات صلة/ مناسبة/ متماسكة/ وصحيحة بدرجة كبيرة

**كمية المعلومات: هل كمية المعلومات المقدمة مرتبطة بمجال التطبيق؛ وهي شاملة لكن موجزة؟**

غ/م لا تتوافر معلومات ضمن التطبيق

1. محدودة أو كثيرة جدا
2. غير كافية أو يمكن أن تكون مبالغة
3. كافية لكنها ليست شاملة أو موجزة
4. تقدم مجالا واسعا من المعلومات، تحوي بعض الثغرات أو تفاصيل غير ضرورية؛ أو لا روابط لمعلومات أو مصادر إضافية
5. شاملة وموجزة؛ تحوي روابط لمعلومات ومصادر إضافية

**المعلومات البصرية : هل العرض البصري للمفاهيم – عبر الجداول/ رسوم بيانية/ صور/ فيديو، إلخ.،** **واضح، منطقي وصحيح ؟**

غ/م لا معلومات بصرية في التطبيق ( مثال، تحوي فقط على صوتيات، أو نص مكتوب )

1. غير واضحة تماما/ محيرة/ خاطئة أو صحيحة ولكن ناقصة
2. بشكل عام غير واضحة/ محيرة/ خاطئة
3. مقبولة، لكن في معظم الأحيان تكون غير واضحة/ محيرة/ خاطئة
4. بشكل عام واضحة/ منطقية/ صحيحة مع بعض المشاكل المحدودة
5. واضحة/ منطقية/ صحيحة بشكل تام

المصداقية: هل للتطبيق مصدر شرعي ( محدد في وصف متجر التطبيقات أو ضمن التطبيق نفسه ) ؟

1. المصدر محدد لكن شرعية المصدر/ موثوقيته مشبوهة ( مثال: مشروع تجاري يحقق منافع)
2. يبدو أنه آت من مصدر شرعي، لكنه لا يمكن التأكد ( لا وجود لموقع إلكتروني)
3. تم تطويره من قبل منظمة غير حكومية صغيرة/ مؤسسة ( مستشفى/ مركز، إلخ) / مشروع تجاري متخصص، جهة مانحة
4. تم تطويره من قبل حكومة، جامعة، أو أي مما ذكر أعلاه لكن ضمن نطاق واسع
5. تم تطويره من قبل جهة حكومية وطنية تنافسية أو ممولة للأبحاث

الإستناد إلى الدليل : هل تم تجربة هذا التطبيق أو اختباره؛ يجب التأكد من صحته بالاستناد إلى الدليل (بحسب دراسات علمية منشورة) ؟

غ/م لم يتم تجربة التطبيق أو اختباره

1. تشير الأدلة إلى أن التطبيق لا يعمل
2. تم تجربة التطبيق ( مثال، مدى قبوله، سهولة الاستخدام، تقييم الرضى ) وحصل على نتائج إيجابية بشكل جزئي في دراسات لم تكن تجارب غير عشوائية وموجهة، أو كان هناك دليل محدود على التناقض
3. تم تجربة التطبيق ( مثال، مدى قبوله، سهولة الاستخدام، تقييم الرضى ) وحصل على نتائج إيجابية في دراسات لم تكن تجارب غير عشوائية أو موجهة، ولم يكن هناك دليل على التناقض
4. تم تجربة التطبيق والنتائج تم اختبارها في 1-2 دراسات عشوائية وموجهة ما يشير إلى نتائج إيجابية
5. تم تجربة التطبيق والنتائج تم اختبارها في أكثر من 3 دراسات عشوائية وموجهة ذات جودة عالية ما يشير إلى نتائج إيجابية

د. متوسط مقياس المعلومات =

* يجب عدم احتساب الأسئلة التي تم تقييمها ب غ/م

**جودة التطبيق من وجهة نظر المستخدم**

**القسم E**

**هل توصي بهذا التطبيق لأشخاص قد يستفيدون منه ؟**

1. بالتأكيد **كلا لن أوصي بهذا التطبيق لأي أحد**
2. **هناك قلة من الأشخاص الذين يمكن أن أوصيهم بهذا التطبيق**
3. **ربما هناك عدة أشخاص يمكن أن أوصي لهم بهذا التطبيق**
4. **هناك العديد من الأشخاص الذين يمكن أن أوصي لهم بهذا التطبيق**
5. **بالتأكيد نعم أنا أوصي بهذا التطبيق للجميع**

ما عدد المرات التي تظن أنك ستستخدم فيها هذا التطبيق خلال الأشهر 12 إن كان مناسباً لك

1. ولا مرة
2. 1-2
3. 3-10
4. 11-50
5. أكثر من 50

هل ستدفع مقابل حصولك على هذا التطبيق ؟

1 كلا

1. ربما
3. نعم

ما هو تقييمك العام لهذا التطبيق بحسب النجوم ؟

1. * واحد من أسوأ التطبيقات التي استخدمتها
2. **
3. *** متوسط
4. ****
5. ***** واحد من أفضل التطبيقات التي استخدمتها

احتساب النتيجة

أ: متوسط مقياس الجذب ...........................................

ب: متوسط مقياس الوظيفية = ...............................................

ج: متوسط مقياس الجماليات= .............................................

د: متوسط مقياس المعلومات = .............................................

النتيجة الوسطية لنوعية التطبيق=...........................................

النتيجة الوسطية لجودة التطبيق الذاتية=......................................

خاص بالتطبيق

هذه البنود الإضافية يمكن تعديلها واستخدامها لتقييم الأثر الملحوظ للتطبيق على مدى معرفة المستخدم، ومواقفه، ونواياه تجاه التغييرومدى احتمال حصول تغيير حقيقي في السلوك الصحي المستهدف.

**القسمF**

**الوعي : من المرجح أن يساهم هذا التطبيق في زيادة الوعي بأهمية التعامل مع (ضع السلوك الصحي المستهدف)**

**لا أوافق بشدة أوافق بشدة**

**المعرفة: من المرجح أن يساهم هذا التطبيق في زيادة المعرفة/ الفهم ل (ضع السلوك الصحي المستهدف)**

**المواقف: من المرجح أن يساهم هذا التطبيق في تغيير المواقف تجاه تحسين (ضع السلوك الصحي المستهدف)**

**نية التغيير: من المرجح أن يساهم هذا التطبيق في زيادة النوايا/ الدوافع على التعامل مع (ضع السلوك الصحي المستهدف**

**طلب المساعدة : من المرجح أن استخدام هذا التطبيق سيشجع على طلب المساعدة من أجل (ضع السلوك الصحي المستهدف) (إن كان هناك حاجة)**

**تغيير السلوك : من المرجح أن استخدام هذا التطبيق سيزيد/ سيقلل من (ضع السلوك الصحي المستهدف)**
